# Supplementary material for: Patterns of Intron Gain and Loss in Fungi
Source: PLoS Biol. 2004 Nov 30;2(12):e422. doi: 10.1371/journal.pbio.0020422 (PMC532390; doi:10.1371/journal.pbio.0020422)
Supplement: Table S1 — Also available at http://genes.mit.edu/NielsenEtAl/. (4.3 MB ZIP). [file pbio.0020422.st001.zip › NielsenEtAl/html/194.html]

AN0575.1.NCU03364.1.MG02398.1.FG05179.1


```
 CLUSTAL W (1.82) Multiple Sequence Alignments - Introns Inserted


Sequence 1: MG02398.1	586 aa
Sequence 2: FG05179.1	905 aa
Sequence 3: NCU03364.1	917 aa
Sequence 4: AN0575.1	902 aa
Alignment Length: 942 aa
Number Identitical Residues: 336 aa
Alignment Score (without introns) 20394


MG02398.1 	------------------------------------------------------------
NCU03364.1	MMQSLKTHASFQRPRTSERDVGSVARDQ-ADVNPAIPSRSSSLHSRITQPMPNSLSGKPQ
FG05179.1 	MPPSLNTHSSFTRPRTSDRDGRPSTRDQGPDQNLLIPSRTSSLHSRITQPIPSTLNMKPQ
AN0575.1  	MGASLRDHS--FRPPTRDR---PSTRDQ-GENSLVVPSRTSSLHSRITQPIPSQSNAKPA
          	   :    :    . : .    . : ..  . .   .: :::  :  :.. ..  . .. 

MG02398.1 	------------------------------------------------------------
NCU03364.1	QRTPKTLTHAYMVCGVGREPSQWVKAPAPAQGKIGHMKGAVGQFWLPEILGSSPRLEQDN
FG05179.1 	QRTPKTLTHAYMVCGVGREPSQWVKAPTPAQGKIGHMKGAVGQFWLPEILGSSPRLEQDN
AN0575.1  	QRTPKTLTHAYMVCGVGREPAQWVKAPTPSQGKIGHMKGAVGQFWLPEILGSSPRLEQDN
          	. :..: : :   .. . ..:.  .:.:.:... .  ..: ..   ..  .::.  ....

MG02398.1 	-----------~------------------------------------------------
NCU03364.1	DIARALHNAMR0ACFPHDVEICTGRSQPHCVHHAFVLQQDSSHTLYGICLRVWSRADEKR
FG05179.1 	EIARALHSAMR0ACFPHDVEICTGRSQPHCVHHSFVLQQDSSHTLYGICLRVWSRADEKR
AN0575.1  	EIAKALHSAMR0ACFPHDVEICTGKNQPHCVHHAFVLQQDSSHTLYGIALRVWSRADEKR
          	. : :  .:   :. . . . .:. ... .   :   ...:: :  . .    : :... 

MG02398.1 	------------------------------------------------------------
NCU03364.1	AETIRELRRRTEPDFFDNPDEQYWIPYCLSFLSRYPLYNLLGDYLRGMWIHWNKATNLFH
FG05179.1 	AETIRELRKRTESDYYDNPDETYWIPYCLSFLSRYPLYNLLGDYLRGMWIHWNKATNLFH
AN0575.1  	AETIRELRKKTESDYYDNPEETYWIPYCLSFLSRYPLYDLLGDYLRGMWIHWNKATNLFH
          	:.:  .    :...  .....    . . :  :  .  .  ..   .     ..::.   

MG02398.1 	------------------------------------------------------------
NCU03364.1	AEEVSRILSFPAPRLNDLVRIDMKDYALCYQFPSSPTGFQNFAMWPLFNCLSIPNIVGVI
FG05179.1 	AEEVSRILSFPAPRLNDLVRIDMKDYALCYQFPSSPTGFQNFAMWPLFNCLSIPNIVGVI
AN0575.1  	AEEVSRILSFPAPRLNDLVRIDMKDYALCYQFPSSPTGFQNFSMWPLFTCLSIPNIVGVI
          	:.. :   : .:.  ..    . .. : . . .::.:. .. :  .  .. : ..  .  

MG02398.1 	---------------------MAAETVRYCVRVYEWSGLYVPMVHARHAKELVQEPGPYI
NCU03364.1	EAAISPTRRIIFVSHYPAMLTMAAETVRFAVRVYEWSGLYVPIVHARHATQLVEEPGPYI
FG05179.1 	EAAISPTRRIIFVSHYPAMLTMAAETVRYCVRVYEWSGLYVPVVHARHAKELVQEPGPYI
AN0575.1  	EAAVSPTRRIIFVSHYPAMLTIAAETIRYCVRVYEWSGLYVPVVHARHIKDLVQEPGPYI
          	.:: :.:      :  .:  ::****:*:.************:***** .:**:******

MG02398.1 	LGVSAECRSLFTAPTDALVVDLDRNFVLTSSPPTALNPNQRNKFVTRLTQALNGDVTPSG
NCU03364.1	LGITAECRSLFTPPADALVVDLDRNFVLTSSPPNALSPSQRNKFVTRLTQALNGDVTPSG
FG05179.1 	LGITVECRSLFTAPTDALVVDLDRNFVLTSSPPTALNPGQRNKFVTRLTQALNGDVTPSG
AN0575.1  	LGVTAECRTLFNAPSDALVVDLDRNFVLTSSPPNILNPGQRTKFINRLTQALNGDVSPSG
          	**::.***:**..*:******************. *.*.**.**:.**********:***

MG02398.1 	APAHLRSAYSGGKVVPAGQIIVMRGEVESIQDPEWWNQDAVMAVMDHVCEKM~GRNTGIK
NCU03364.1	VPPHLRSAYGGGKLIPAGQIIVMRGEVESIQDPDWWNQDAVMSVMDHVCEKM~GRNTGIK
FG05179.1 	VPQHLRSAYGGGKLVPAGQIIVMRGEVESIQDPEWWNQDAVMAVMDHVCEKL~GRNTGIK
AN0575.1  	VPNHLRSAYAGGKLIPAGQIIVMRGEVESVEEPSWWNQDAVMGVMDHVCEKL0GRNTGMK
          	.* ******.***::**************:::*.********.********: *****:*

MG02398.1 	AVFGGAVKKPLMTKVSMRHLNEIVRERNQYSRDALEAWQDFINLKGRMDTELAKVTKRNN
NCU03364.1	AVFGGSAKKPLMTKVSMRHLNEIVRERNQYSRDAMEAWQDFINLKGRMETELSKVTKRNN
FG05179.1 	AVFGGSVKKPLMTKVSMRHLNELVRERNQYSRDALEAWQDFINLKGRMDTELSKVNKRNN
AN0575.1  	AIFGGSVKKPLMTKVSMRHLNEIVRERNQYSRDAMEAWQDFINLKGRMDTELGKVTKRNN
          	*:***:.***************:***********:*************:***.**.****

MG02398.1 	YLNEELDSWKQQ0FTKFQAFAEQLTKETQDLKVKIEGHKRENRRLATLIDQQKDDAARMS
NCU03364.1	YLNDELESWKQQ~FLKFQTFAETLTKETQDLKAKIENSKRENRRLGSLIEEHRSDYNRLQ
FG05179.1 	YLVEELESWKQQ~FLKFQAFAETLTKETQDLKVKIDTHKRENRRLAGLIDQQKDDNARLS
AN0575.1  	FLVEELETWKQQ~FLKFQAFAEQLTKETSELKVKIENHKRENRRLSSLIDQQKDDVARLT
          	:* :**::**** * ***:*** *****.:**.**:  *******. **::::.*  *: 

MG02398.1 	VRLTGTEKQRDDALEALVLQQEIAEELERERKRNKKELAALQHTNHTITRQRDDARRVVL
NCU03364.1	MRLQGTEKQRDDALEALVLQQEIAEELERERKRNRKELTALQHTNNTITRQRDEARRVVL
FG05179.1 	VRLTGTEKQRDDALEALVLQQEIAEELERERKRNKKELSQLQHTNVTIMRQRDEARRVVL
AN0575.1  	LRLSGTEKQRDDALEALVLQQEIAEELERERKRNQKELAALQHTSATLARQRDDAQRVVL
          	:** ******************************:***: ****. *: ****:*:****

MG02398.1 	HLRSLIGGQSHHLEHLVGSLTKPENDNDLAHEVEAGYEDDTQD1MESKHH~N--------
NCU03364.1	HLRSLISGQHHHMEHIVKTMTSPQ---ELAAEIEAGFVAAAQE~AEEATA1GE-------
FG05179.1 	HLRSLIGGQSHHMEHLIQSLTKPD---DLAQEIEEGYDETEEE~TQSSEP~SRL------
AN0575.1  	HLRSLINGKSHHMEHLVRSIGSSAELDELAEQEVAEEQAQESA~ESVKDK~NVKKDVKTN
          	******.*: **:**:: :: .. . .:** :         .   .     .  .. .:.

MG02398.1 	-----------------------SKRFSSSSFSDVADRHLKDKTDAIAHIIRNIAEQCQA
NCU03364.1	---------EPNSERFIKNLTLASKRISSQTFVDVADRHLKDKTDAIAHIIRNIAEQCQA
FG05179.1 	---------TPSPSPTTTSSTSRSKRYSSASFSDVADRHLKDKTDAIAHIVRNIAEQCQA
AN0575.1  	ESSNGVSSINPDLEQHLLNIGQPERRLARLSVTDVADRYLRDKTDAISEIIRSISEQCAA
          	.::.. ::  .. .    .    .:* :  :. *****:*:******:.*:*.*:*** *

MG02398.1 	AVEGLALAHDAEIEDGSDSGHHS-AHHSSSYRRGSGLSVAGSDAGTH---SEVGDDISVR
NCU03364.1	AVDGLQLAQDAEIGTHHEPLSAKGAGMYSKSRRGSNLSIAHSDDGTSVATTEAGDDS-FL
FG05179.1 	AVEGLQLAHDAEL--GSSSRRNS-RHSTIQSDDG--------HSAAT---SETGDES--L
AN0575.1  	AVEGLQLAQDAEDGDAMPSKANLEPRGMMTPREG----------------SEMGDSEVST
          	**:** **:***      .              *                :* **.    

MG02398.1 	LRPSSGRTSSIPPTPDLIPNRSSTALSMASTTMTTPERSSQQYMIG-HDIPTKIVEDDED
NCU03364.1	HPGASGRVSSIPPTPDLIPNRSSTSMSIAS-TATTPERTSQQYSLGHHDIPTRILEDDED
FG05179.1 	LAPRSGRASSIPPTPDLIPNRSSTAMSFAS-TATTPERSSQQYSLR-DEIPTKIVEDDEE
AN0575.1  	LHP-DGRTS-VPPTPDLVHNRSSTSMSMIS-SSTFPERSSQQYGPG--EIPTRIVEDDDE
          	    .**.* :******: *****::*: * : * ***:****     :***:*:***::

MG02398.1 	LDNGPSASESETVNMPAHTTSRVLGKHTESLLHRPSGARISALGGTR
NCU03364.1	---ELAGERSESENPIPHEPSNFVGKHAESLIHRPSGARISALGSSR
FG05179.1 	-DFEENRSDNGTVSHETSVVSKHQLQHQQSLMHRPSGARISALGGTR
AN0575.1  	-------HAHETEGLDNPSEAGTLSKHSNEDLMRTTPRMLA------
          	           : .      :    :* :. : *.:   ::
```
